# Spherical nucleic acids: Organized nucleotide aggregates as versatile nanomedicine

**DOI:** 10.1002/agt2.120

**Published:** 2021-09-14

**Authors:** Yangmeihui Song, Wenyu Song, Xiaoli Lan, Weibo Cai, Dawei Jiang

**Affiliations:** 1 Department of Nuclear Medicine, Union Hospital, Tongji Medical College, Huazhong University of Science and Technology, Wuhan, China; 2 Hubei Key Laboratory of Molecular Imaging, Wuhan, China; 3 Departments of Radiology and Medical Physics, University of Wisconsin-Madison, Madison, Wisconsin, USA

**Keywords:** drug delivery, gene regulation, molecular imaging, spherical nucleic acids

## Abstract

Spherical nucleic acids (SNAs) are composed of a nanoparticle core and a layer of densely arranged oligonucleotide shells. After the first report of SNA by Mirkin and coworkers in 1996, it has created a significant interest by offering new possibilities in the field of gene and drug delivery. The controlled aggregation of oligonucleotides on the surface of organic/inorganic nanoparticles improves the delivery of genes and nucleic acid–based drugs and alters and regulates the biological profiles of the nanoparticle core within living organisms. Here in this review, we present an overview of the recent progress of SNAs that has speeded up their biomedical application and their potential transition to clinical use. We start with introducing the concept and characteristics of SNAs as drug/gene delivery systems and highlight recent efforts of bioengineering SNA by imaging and treatmenting various diseases. Finally, we discuss potential challenges and opportunities of SNAs, their ongoing clinical trials, and future translation, and how they may affect the current landscape of clinical practices. We hope that this review will update our current understanding of SNA, organized oligonucleotide aggregates, for disease diagnosis and treatment.

## 1 | INTRODUCTION

Nanomedicine is defined as the medical application of nanotechnology. It utilizes structures’ physical and chemical characteristics on a scale similar to biomolecules and biosystems, ranging from single to hundreds of nanometers for diagnostic and therapeutic purposes.^[1]^ With the ever-increasing progress of chemistry and biology in drug discovery, researchers have been advancing towards systematic understanding and comprehensive manipulation of nanomedicine’s biological profiles in living organisms.^[2]^ It is researchers’ general belief that well-designed molecules in highly controllable forms of nanomaterials will exhibit programmable biological properties for disease imaging and treatment.^[3]^ Compared with endogenous macromolecules, nanomedicine presents excellent controllability concerning their shape, size, surface chemistry, and loading capacity and may harbor multifunctional components for synergistic biomedical applications, such as drug delivery, gene delivery, bioimaging, and disease treatment.^[4]^

Oligonucleotides carry, transfer, and edit genetic information when used as therapeutics for various diseases via gene therapy, antivirus treatment, and, most recently, COVID-19 vaccination.^[5]^ However, rapid excretion in vivo and suboptimal stability limit their further biomedical applications. As such, researchers have been making efforts to establish nanoscale drug delivery systems for deoxyribonucleic acid (DNA) or ribonucleic acid (RNA). In 1996, Mirkin and coworkers first described an organized self-assembly strategy to obtain SNA through grafting DNA oligonucleotides onto the surface of Au nanoparticles (AuNPs) for macroscopic material preparation and nucleotide delivery.^[6]^ Giving new entry into this new class of DNA/nanoparticle hybrids, the SNA presents a versatile tool for mediating nanoparticle bio interfacing with living organisms. At the same time, the physiochemical properties of SNA (such as size, shapes, and composition) define their distinctive biological profiles in vivo. Later, after the use of quantum dots (QDs) as an SNA core for DNA grafting in 1999, a variety of SNAs with various core particles and surface oligonucleotides emerged, leading to the generally accepted concept of SNAs as “organized nucleotide aggregates on the surface of nanoparticle cores”.^[7]^

SNA’s three-dimensional architecture imparts intriguing physical and chemical properties, making them superior to linear forms of nucleic acids in biomedical applications. Research on SNA has revealed that the core particles underlie its physicochemical characteristics (such as plasmonic, catalytic, and optical properties) that are particularly important in the design of the nanosystem.^[8]^ On the other hand, the DNA shell facilitates special molecular recognition with a binding affinity a hundred times higher for complementary sequences comparing with their linear counterparts.^[9]^ As such, accurate interactions with target molecules are achieved for biosensing, bioimaging, and drug/gene delivery. Superior to linear DNA, SNA can penetrate many biological barriers (such as the epidermal, blood–brain, and blood–tumor barriers) and shuttle nuclear acids into cells or tissues without adding ancillary transfection agents or concerns on nuclease degradation.^[10–13]^ Furthermore, when comparing with other gene delivery systems such as liposomes, SNA presents good immunogenicity.^[14,53]^ Therefore, they have been evolving into versatile tools as delivery vehicles of nucleic acids, drugs, and proteins for molecular diagnosis, gene regulation, drug delivery, immune-modulation, among other biomedical applications.

**FIGURE 1:**
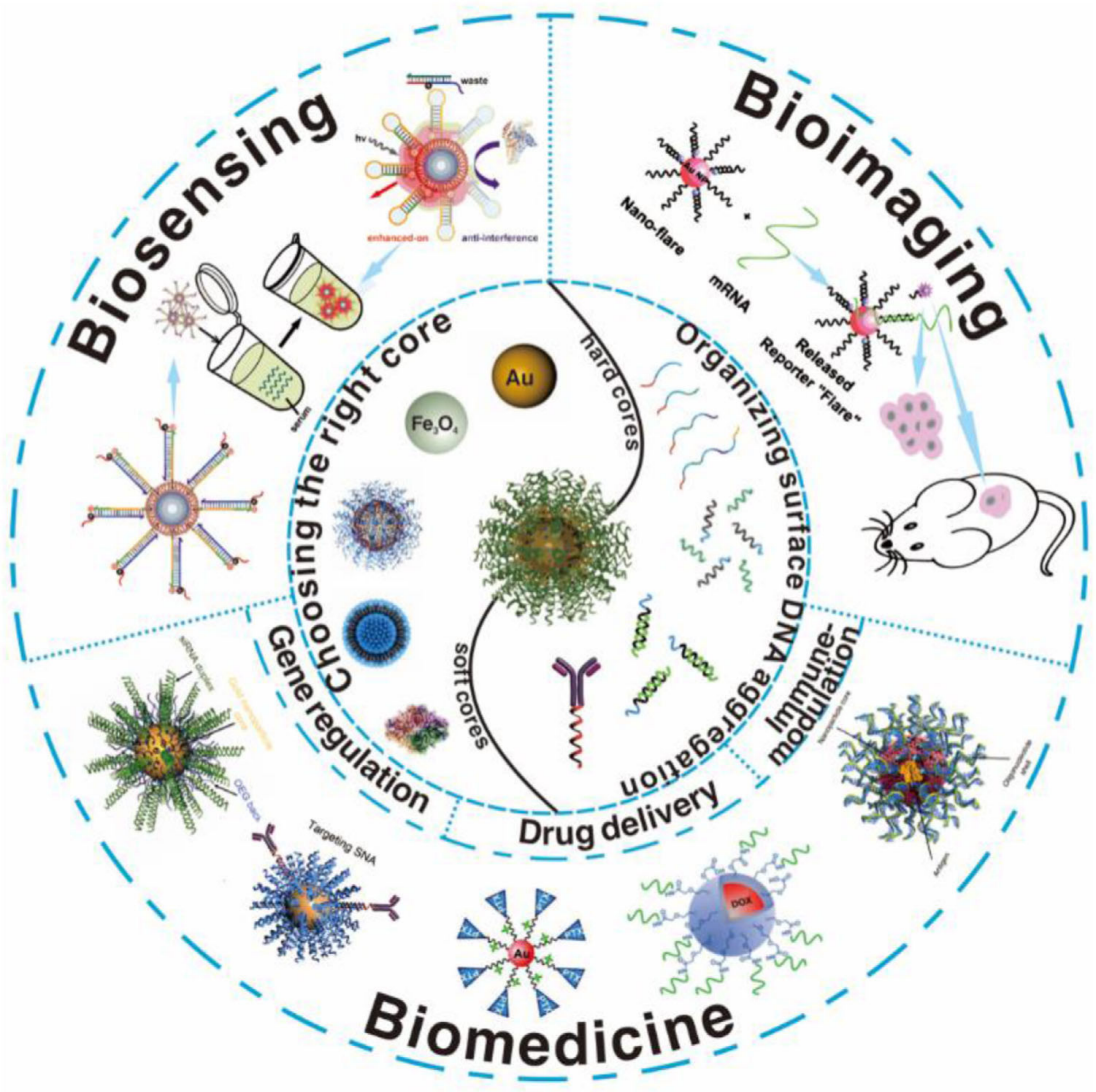
Representative examples of SNA structures from a nanoparticle core and a layer of densely arranged oligonucleotide shell. SNA’s three-dimensional architecture imparts intriguing physical and chemical properties and evolves them into versatile tools as delivery vehicles of nucleic acids, drugs, and proteins for molecular diagnosis, gene regulation, drug delivery, immune-modulation, and other biomedical applications. Reproduced with permission: Copyright 2012, American Chemical Society.^[8]^ Copyright 2007, American Chemical Society.^[15]^ Copyright 2014, American Chemical Society.^[16]^ Copyright 2015, American Chemical Society.^[17]^ Copyright 2011, American Chemical Society.^[18]^ Copyright 2021, The American Association for the Advancement of Science.^[19]^ Copyright 2019, Elsevier.^[158]^ Copyright 2011, American Chemical Society.^[21]^ Copyright 2017, American Chemical Society.^[22]^ Copyright 2019, Springer Nature^[23]^

This review presents an overview of recent progress on SNA to speed up their biomedical application and potential translation into the clinic. We start with introducing the concept and characteristics of SNAs as drug/gene delivery systems with respect to the core materials and the surface nucleic acid assembly and highlight recent efforts of bioengineering SNA for imaging and treatment of various diseases (Figure 1). We further discuss the challenges and opportunities of SNA for theranostics, their potential clinical translation, and how they impact the current landscape of clinical practices. We believe that this review may update our current understanding of SNA, an organized oligonucleotide aggregate, for disease diagnosis and treatment.

## 2 | CHOOSING THE RIGHT CORE

To successfully deliver nucleic acids using SNA, choosing the right nanoparticle core plays an essential role. This directly determines the shapes, sizes, and biological profiles of the core–shell assemblies. Inorganic nanomaterials, such as gold/silver/Pd/Pt nanoparticles, QDs, or iron oxide nanoparticles, have all been used for ordered DNA/RNA grafting on their surfaces.^[6,7,24–26]^ Subsequently, organic nanomaterials in the forms of liposomes, polymers, and even proteins, have been employed to obtain more biocompatible SNAs.^[16,17,27,28]^ In this section, we go through the most commonly used SNA core materials and introduce how they have been engineered for dense nucleic acid coatings.

### 2.1 | Inorganic cores

**FIGURE 2:**
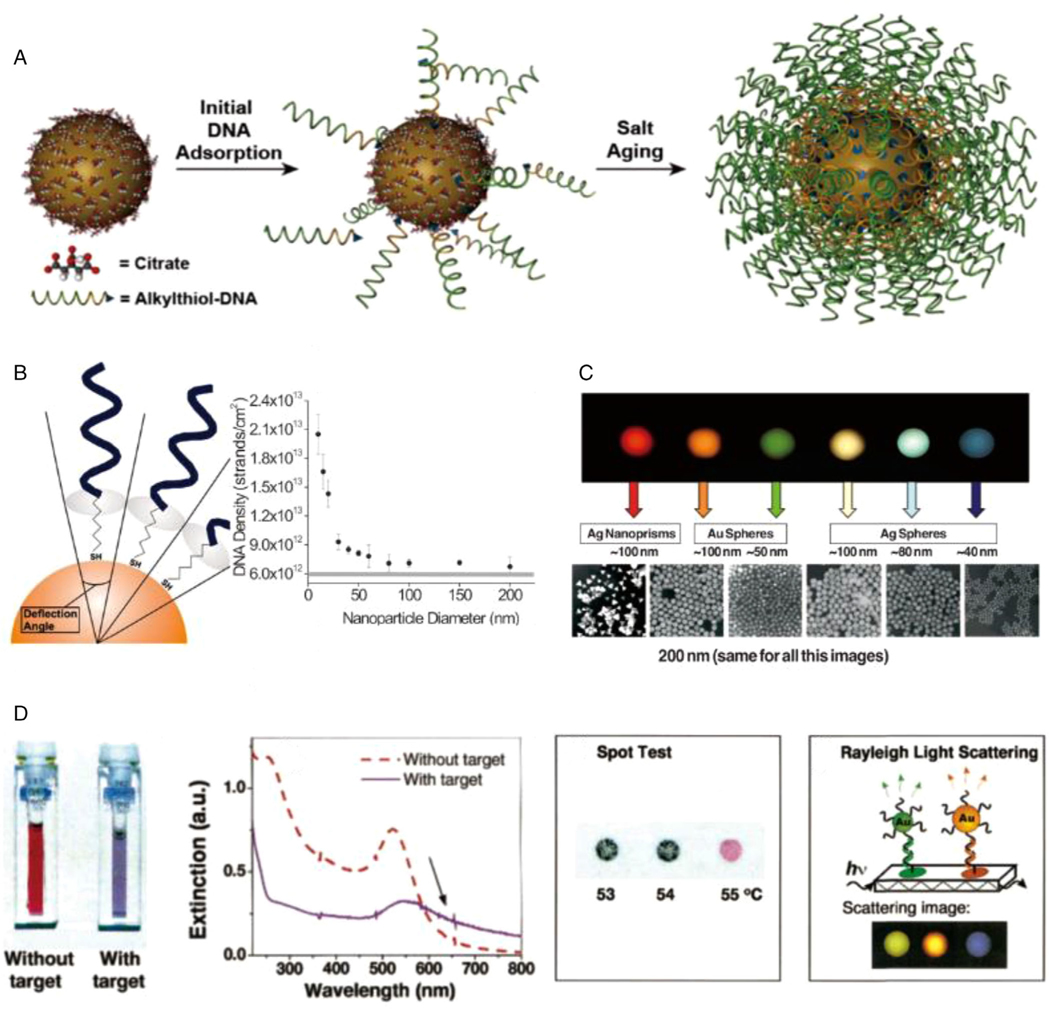
(A) Synthesis paradigm of SNA–AuNP conjugates. Citrate-stabilized nanoparticles were used as cores, playing supporting roles by incubating with alkyl thiol-functionalized oligonucleotides in aqueous solutions of successively high concentrations of salt and surfactant over ~12 h. Reproduced with permission: Copyright 2012, American Chemical Society.^[8]^ (B) Oligonucleotide density is determined as a function of AuNP diameter. Reproduced with permission: Copyright 2009, American Chemical Society.^[32]^ (C) A metal nanoparticle’s size, shape, and composition can be systematically varied to produce materials with distinct light-scattering properties. Reproduced with permission: Copyright 2005, American Chemical Society.^[33]^ (D) Colorimetric response, UV–vis spectra, *T_c_* (thermal shift associated with color change) of target-linked AuNP aggregates. Rayleigh light scattering from nanoparticles colorimetric response of different sizes and compositions on a glass chip: green, Au 50 nm; orange, gold 100 nm; and purple, silver 40 nm. Reproduced with permission: Copyright 2003, American Chemical Society.^[34]^ Copyright 1997, The American Association for the Advancement of Science^[35]^

Noble metal nanoparticles have been widely applied as core materials for SNA preparation, the most representative of which are AuNPs. AuNPs can be prepared via accessible synthetic methods to obtain a wide range of particle diameters, allowing direct control over the shape and size of the final SNA products.^[29]^ In 1996, Mirkin and colleagues established a paradigm of preparing gold core SNAs by capping 13 nm AuNPs with thiol-DNA for colloidal aggregation in a rational and thermally reversible manner (Figure 2A).^[6,8]^ This strategy expanded our toolbox of tailoring nanoparticle aggregation, with the help of densely coated DNA, to modulate their optical, electronic, and biological properties.^[30]^ In 2015, the Chan group constructed core–satellite superstructures with stable tumor accumulation and improved body elimination based on the strategy.^[31]^ The core–satellite design employs DNA as the key to control the AuNP superstructure delivery and metabolism by orchestrating AuNPs into colloidal materials. With individual AuNP as building blocks, the DNA-regulated core–satellite structure presents highly controllable sizes, surface chemistry, and final architecture. They interact with cells and organs/tissues as a function of their structural design, with the degradation of DNA bridge marking the disassembles of the superstructure, enabling their escape from biological sequestration. The high surface area of AuNP also allows for the dense presentation of multifunctional components, enabling advanced drug/gene delivery for therapeutic purposes.

Besides gold, a silver nanoparticle (AgNP) is a valuable candidate for DNA grafting. Oligonucleotides with multiple cyclic disulfide groups can link with silver atoms and prevent Ag from being oxidized.^[24]^ Alternatively, silver nanoprisms can be functionalized with DNA through a monolayer of silica coating.^[36]^ Densely functionalized iron oxide nanocrystals (Fe_3_O_4_ nanoparticles) with a surface DNA coating allow for a myriad of applications, such as magnetic resonance imaging (MRI), magnetothermal therapy, and magnetic structure assembly for electronic memory.^[37]^ Semiconductor nanocrystals present an exclusive quantum confinement effect, adding designable electronic energy states and tunable optical transitions to SNAs, whose light emission covers the ultraviolet, visible, near-infrared, and mid-infrared spectral ranges.^[38]^ This contributes to the great promise of semiconductor nanoparticles as imaging probes, biosensors, catalysts, and bio-labeling materials.^[39]^ More types of SNAs can be prepared from metal nanoparticles with hydrophobic capping ligands, including Pt, Al, Pd, Cu, Co, In, Ni, and their mixtures.^[40,41]^

The shape and size of core materials directly dictate the maximum oligonucleotide grafting density on the particle surface.^[42]^ Given that the diameter of a DNA double-strand is approximately 2 nm, preparing SNAs on spherical metal cores can be simplified as wrapping a solid sphere with 2 nm-sized tapes.^[43]^ Following this thought, shape affects the DNA coating density: a 10-nm-sized spherical AuNPs may support 3.5 times more oligonucleotide strands than a planar gold plate with the same surface area. Size is also important as it determines the radius of curvature: smaller AuNPs present a higher curvature, providing a natural deflection angle for increased nucleotide grafting (Figure 2B).^[32]^

In addition to controlling nucleotide presentations on the surface, solid SNA cores present tunable optical features and durable catalytic stability (Figure 2C).^[33]^ Upon binding with different analytes, various types of nanoparticles in the SNA center bring surface-enhanced Raman scattering, surface plasmon resonance (SPR), and sensitive redox activity. They lead to sensitive biosensing, signal augmentation, and energy transformation in response to microenvironmental changes for effective biomedical applications (Figure 2D).^[34,35,44]^

### 2.2 | Organic core

Although inorganic nanoparticles are most commonly used to prepare SNAs, they bring potential long-term toxicity concerns due to the tendency to accumulate in organs such as the liver and spleen.^[45]^ After SNAs enter cells via endosomes during incubation, oligonucleotide fragments are often phased back out of the cell while the nanoparticle cores are retained.^[46]^ Therefore, recently developed biodegradable and biocompatible nanostructures, such as liposomes, proteins, and poly(lactic-*co*-glycolic acid) (PLGA), have opened up exciting new avenues for biomedical applications of SNAs.

#### 2.2.1 | Liposome

**FIGURE 3:**
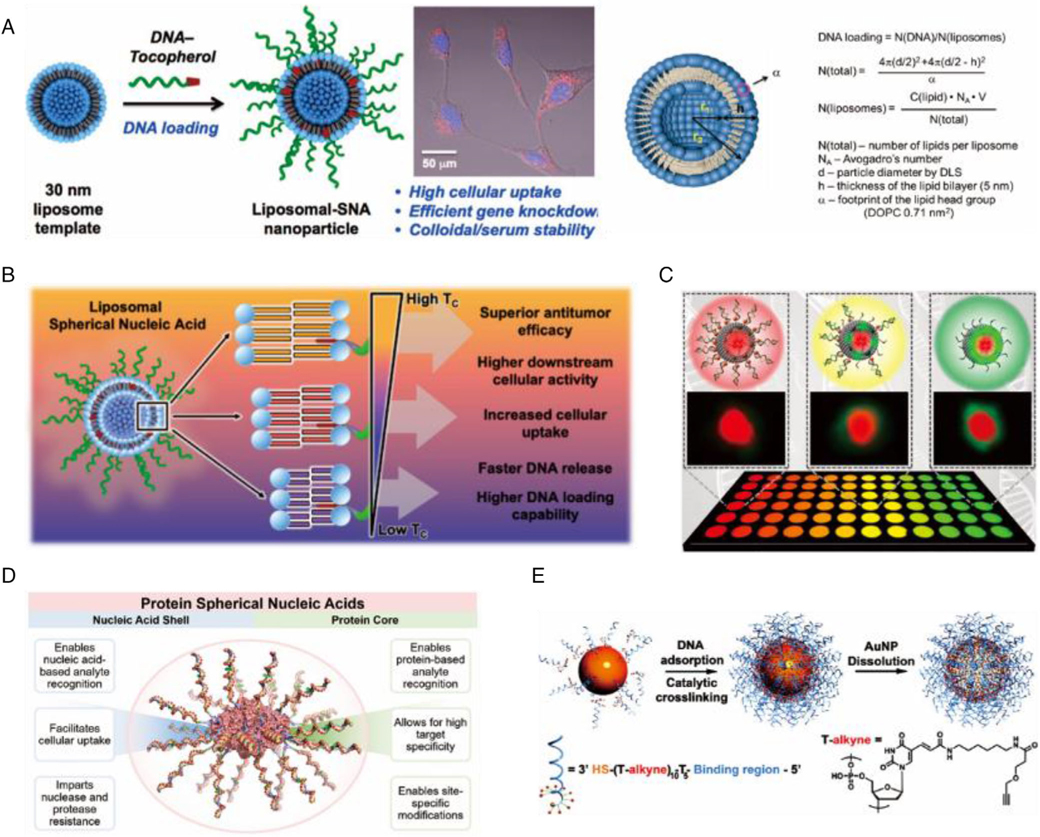
(A) The surface of the liposomes was functionalized with DNA strands modified with a tocopherol tail that intercalates into the phospholipid layer of the liposomal core via hydrophobic interactions. Reproduced with permission: Copyright 2014, American Chemical Society.^[16]^ (B) The constituent lipids determine the biological and immunological properties. Reproduced with permission: Copyright 2021, American Chemical Society.^[47]^ (C) LSNA-nanoparticle hybrids containing either QDs or gold nanoparticles. Reproduced with permission: Copyright 2020, American Chemical Society.^[49]^ (D) A Pro-SNA with functional protein as the core. Reproduced with permission: Copyright 2015, American Chemical Society.^[17]^ (E) A new class of polyvalent nucleic acid nanostructures (PNANs), the first core-free structures with high-density DNA shells. Reproduced with permission: Copyright 2011, American Chemical Society^[18]^

Liposomal SNAs (LSNAs) are a new class of metal-free SNA nanostructures that can be easily synthesized from readily available starting components (Figure 3A).^[16]^ LSNAs are biocompatible and chemically adjustable, generally synthesized by anchoring nucleic acids modified with hydrophobic components, such as cholesterol, to the lipid bilayer of liposomal templates. The lipid constituents determine their thermal stability, which further affects the DNA loading, serum stability, cellular uptake, in vitro immune-activation, and in vivo lymph node accumulation of the LSNA (Figure 3B).^[47]^ LSNAs synthesized using higher phase transition temperature lipids with more stable liposome scaffolds displays improved serum stability and extended blood circulation. The affinity of the DNA shell also affects overall nanostructure stability and the release rate of oligonucleotides from the liposome core. High-affinity diacylglycerol lipid tails provide LSNAs a twofold increase in oligonucleotide loading, resulting in a 20-fold longer circulation half-life and triggering faster cellular internalization with more robust immune activation than cholesterol-based LSNAs.^[48]^

**FIGURE 4:**
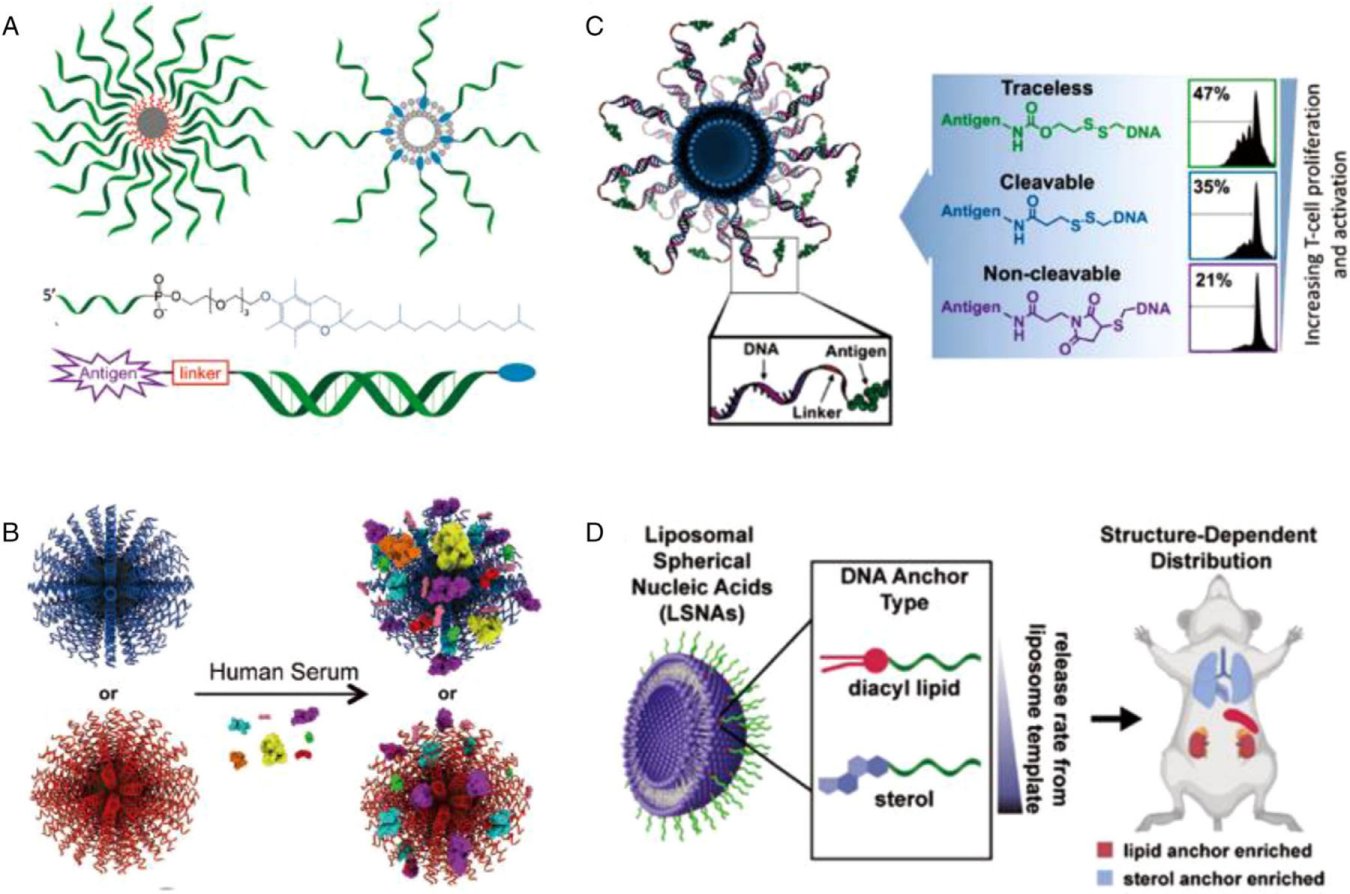
(A) SNAs present nucleic acids as outward-facing spherical arrays formed of a recognition sequence, a spacer, and an attachment group. Reproduced with permission: Copyright 2019, American Chemical Society.^[59]^ (B) Sequence-specific interactions of SNAs and human serum proteins. Reproduced with permission: Copyright 2014, Wiley.^[60]^ (C) SNAs link DNA to antigens via a linker and alter downstream T cell responses. Reproduced with permission: Copyright 2018, American Chemical Society.^[55]^ (D) Two types of LSNAs differ only by the affinity of the modified DNA sequence for the liposome template. Reproduced with permission: Copyright 2020, American Chemical Society^[57]^

In animals, LSNAs are not immediately cleared from the circulation but will guide nucleic acids into the mononuclear phagocyte system, which can be harnessed for effective activation of immune cells, such as macrophages and dendritic cells (DCs).^[50]^ CpG-embedded LSNAs are approximately 80-fold more immune potent than free oligonucleotides, serving as liposome-based immunostimulants and potent cancer immunotherapeutics.^[51–54]^ LSNAs incorporating tumor-associated antigens can be used to modulate the kinetics of antigen presentation and the expression of costimulatory markers to improve antitumor efficacy.^[55]^ The distribution of LSNAs in vivo is influenced by the affinity of DNA to its liposomal template. Cholesterol-based LSNAs exhibit high DNA delivery efficiency to the lung, whereas LSNAs with high-affinity diacylglycerol lipid tails show high DNA accumulation in the kidney, and both of them showed high accumulation in the spleen (Figure 4D).^[56–58]^ As such, LSNAs have the potential to co-deliver drugs and nucleic acids to different significant organs. However, the biological fate of LSNAs remains unknown mainly and demands further investigation.

#### 2.2.2 | Protein

The protein SNAs (Pro-SNAs) formed with a protein core and a dense shell of oligonucleotides via click chemistry is a novel type of SNA. It can be efficiently transported into cells. Mirkin et al. pioneered the covalent attachment of the oligonucleotide shell to the surface of a sizeable homote-trameric enzyme (*β*-galactosidase), which allows simultaneous testing of intracellular delivery and catalytic function. Compared to *β*-galactosidase with four fluorophore modifications, ProSNA *β*-galactosidase showed up to ~280-fold cellular uptake and reduced the enzyme working concentrations to as low as 100 pM (Figure 3D).^[17]^ Compared to a protein-linked hexamethylene glycol, Pro-SNAs promote a sevenfold increase in cellular uptake while maintaining enzymatic activity in vitro.

Furthermore, the formation of pro-SNA from G-quadruplex increased cellular uptake up to four times. When used in animals, regardless of sequence, Pro-SNAs exhibit prolonged circulation and higher accumulation in major organs (including lung, kidney, and spleen) while maintaining enzymatic activity.^[61]^ In Pro-SNAs, the functionalization of proteins by DNA will alter the surrounding ionic environment nonuniformly, which can be identified using solution X-ray scattering and density functional theory.^[62]^ The latest cross-linking strategy is to assemble a single Pro-SNA with a lactate oxidase core into nanoscale particles (namely cross-linked SNA, or X-SNA), which further enhances cellular delivery efficiency and the signal-to-noise ratio of the intracellular sensor of Pro-SNAs.^[63]^ It is foreseen that Pro-SNA holds highly promising for protein-based diagnostic and therapeutic applications ranging from immunotherapy to enzyme replacement therapy.^[64]^

#### 2.2.3 | Polymer

In 2004, a versatile method for preparing novel polymeric DNA amphiphiles by solid-phase DNA synthesis was established. The assembled spherical micelle structures exhibited specific recognition properties defined by their DNA sequences.^[27,65]^ A decade later, DNA-functionalized infinite coordination polymer nanoparticles were designed as biocompatible gene modulators.^[66]^ Subsequently, multiple DNA ligands were grafted onto one end of a polyester chain (polycaprolactone) to generate amphiphilic DNA brush block copolymer (DBBC) structures capable of assembling into spherical micelles in an aqueous solution.^[67]^ Compared to AuNP-based SNAs, DBBC-based micelle SNAs have higher nucleic acid surface density, increased surface negative charges, higher unwinding temperatures, more cooperative thermal denaturation properties, and more efficient transfection-free cellular uptake. The formation of DBBC-based SNAs composed of different polyester units can act as regulators of intracellular biological processes. Of note, micelle-SNAs derived from DNA-DBBCs show effective target gene knockdown in vitro.^[67,68]^

The polymer can be progressively degraded under physiological conditions due to acid-catalyzed or esterase-catalyzed cleavage of ester backbone bonds.^[69]^ To control the stability of nucleic acid coating, a variety of polyesters (e.g., polylactic acid and PLGA) can be used to initiate polymerization reactions.^[70,71]^ The novel SNA using PLGA nanoparticles as the core also exhibits good cellular absorbability and may freely enter macrophages to activate toll-like receptor 9 (TLR-9) in a dose-dependent manner.^[72]^ Pluronic F127 poly(ethylene oxide)–poly(propylene oxide)–poly(ethylene oxide), a Food and Drug Administration (FDA) approved amphiphilic block copolymer, can be assembled into spherical micelles at low critical micelle concentrations at room temperature and used as an effective TLR-9 immunomodulator.^[73]^ A small-sized (~65 nm) polymeric nanoparticle (PNP) containing doxorubicin (Dox) is modified with oligonucleotides to form colloidally stable Dox-containing polymeric SNA (Dox-PSNA) nanostructures.^[22]^ The nucleic acid shell promotes the cellular uptake of Dox-PSNA, leading to increased cytotoxicity against cancer cells.

#### 2.2.4 | Other macromolecules

The amphiphilic self-assembly of nucleic acid–drug conjugates was exploited to generate SNAs with unique properties. They have densely arranged nucleic acids, rapid cellular uptake, and enhanced anti-nuclease stability, making these structures suitable as a carrier-free delivery platform. For example, DNA–camptothecin amphiphile nanostructures display localized light-controlled cytotoxicity, providing a favorable therapeutic window for potential clinical applications.^[74]^ An amphiphilic DNA-paclitaxel conjugate that is stable in solution is tethered to the drug by a biosensing-activated autolytic disulfide linker. It enters the cell and releases the drug at a 100-fold higher rate than free DNA, revealing almost identical cytotoxicity to the free drug. The nucleic acid component serves as a therapeutic payload for intracellular gene regulation and a delivery vehicle for the drug.^[75]^

New metal-organic frameworks were developed based on significant advances in the size and shape of SNA nuclei.^[76]^ A zirconium-based framework, UiO-66-N_3_(Zr_6_O_4_OH_4_(C_8_H_3_O_4_-N_3_), was successfully synthesized and characterized, allowing rapid functionalization of oligonucleotides by Cu-free tension-alkyne click chemistry.^[77]^ A novel class of modular nanostructures was created, providing new programmable atomic equivalents for the assembly strategy of nucleic acids.

DNA nanoflowers and nanoclew, generated from long DNA-building blocks via rolling circular replication, prove that DNAs can also serve as the core of SNAs.^[78,79]^ Other than loading single-stranded DNA or RNA, framework nucleic acids (FNAs) can also be used to form SNAs. Valence-controlled FNA core-based molecular SNAs (FNA-mSNAs) with adjustable biosensor properties (including response dynamics, detection sensitivity, and response range) have been developed.^[80,81]^ FNA-mSNA consists of a DNA nanocube with adjustable valence and a precise number of DNA that can be controlled at each core. Homogeneous FNA-mSNA with different valence can be efficiently designed by simply changing the binding number of the DNA strands.

One novel core-less (hollow) form of SNA has been derived by cross-linking the DNA on the surface of AuNPs and dissolving gold with potassium cyanide (Figure 3E).^[18]^ Composed entirely by nucleic acids, the hollow nanostructures share nearly identical properties of SNA, such as cooperative hybridization of complementary nucleic acids, nuclease resistance, effective gene regulation, and low cytotoxicity. In addition, hollow SNAs can be formulated from silica, another semiconductor material. Comparing with normal SNAs, hollow SNAs offer advanced biocompatibility with improved entry into cells, may avoid unintended effects of uncoated nanomaterials on cellular function, alleviating concerns over their immunogenicity and nucleic acid stability.^[18,46]^

## 3 | ORGANIZING SURFACE DNA AGGREGATION

The densely organized DNA shells of SNAs are generally composed of three parts: a nanoparticle attachment moiety, a spacer, and a programmable functional region (Figure 4A).^[8,59]^ Each component plays an essential role in the functioning of the conjugates. Extensive studies of the various components have provided a valuable toolbox of chemically altered nucleic acids to select for designing and improving the stability, bio-function, and applications of SNAs.

### 3.1 | Attachment moiety

Oligonucleotides can be attached to the nanoparticle core in a covalent or noncovalent manner through a linker moiety. The SNA core material determines what type of attaching moiety can be used for nucleotide coating. As mentioned above, the linking molecule for gold nanoparticles is typically a single propyl or hexyl thiol group, which can be synthesized by conventional phosphoramidation.^[64,73]^ The adsorption of thiols on gold without side reactions results in very high yields. The stability of SNA conjugation is usually determined by monitoring the rate of oligonucleotide substitution by the disulfide reducer dithiothreitol.^[82]^ In addition to the typical salt-aging method, rapid low-pH assisted protocols and freeze–thaw cycle procedures have been employed to modify attachment. A strategy based on the rapid dehydration of the DNA/NP mixture in contact with the butanol phase has been recently reported, greatly accelerating the Au-S bond anchoring reaction on the NP surface.^[83]^ With this strategy, the loading density of DNA is increased approximately three times, and the process can be completed in seconds. Other than Au-thiol conjugation, Fan and coworkers revealed the intrinsic binding between polyadenine (polyA) bases and AuNPs.^[84–86]^ Placing polyA bases at different sections of one DNA strand may yield SNAs with variable valences, mimicking the natural design of carbon atoms for the hierarchical establishment of higher-order structures. Other common attachment groups for inorganic cores are N3, NH2, COO, and chelators such as cyclic disulfides or branched thiol structures. Au–Se bond-based SNA probes were recently obtained by selenol terminal-functionalized molecular beacons and exhibited better antibiotic thiol interference effects, which avoided false-positive signals when imaging biomarkers in living cells.^[87]^ Organic core-linked oligonucleotides often select organic ligands, such as Dibenzocyclooctyne-amine and tocopherol moiety.^[61,67,88,89]^

### 3.2 | Spacer region

Spacers are responsible for placing recognition regions on the NP surface to prevent salt-induced aggregation, allowing greater flexibility, stability and increasing oligonucleotide loading density of conjugated oligonucleotides.^[90]^ The spacer regions usually consist of DNA bases, such as 10 thymine/alanine sequences or polyethylene glycol (PEG) units.^[91,92]^ Increased PEGylation has been reported to extend the circulating half-life of SNAs in mice resulting in reduced cellular uptake.^[93]^

### 3.3 | Recognition sequence

The outermost free component is the recognition portion of the functionalized nucleotide for base pairing with other strands of interest. This section is tailored for specific functional applications, such as ligands with sticky ends, the target strand for the detection or delivering, the complementary strand for siRNAs in gene regulation. Rosi et al. first demonstrated that SNAs could enter cells and regulate gene expression using single-stranded DNA.^[94,95]^ Since then, double-stranded DNA, siRNA, or miRNA conjugates have been applied to SNAs.^[92,96–98]^ The aggregation of surface oligonucleotides will determine SNA’s biological behaviors from the moment they interact with cells in vitro or organs in vivo.^[99,100]^ Mirkin and coworkers showed that by simply changing the nucleic acid sequences, SNA’s protein corona presented a significant change (Figure 4B).^[60]^ Furthermore, when surface encapsulated using antigen conjugated nucleic acids, SNA can be used for immuno-activation via T cell training (Figure 4C).^[55]^

#### 3.3.1 | DNA or RNA

The recognition portion can be made up of any unit chemically bonded by phosphamide, the simplest form of which is conventional nucleic acids (DNA or RNA).^[8]^ The number of oligonucleotides covering the surface of the nanoparticle can be predicted as a function of curvature, shape, and available surface area. The oligonucleotide sequences are designed to complement the target (protein, other linked sequences, reporter sequence, miRNA). On this basis, the ends can be attached to carriers such as imaging agents (fluorescein, radionuclide ^19^F, MRI contrast agents Gd^3+^, drugs (PtII, paclitaxel), and immunostimulants (antibodies, antigens, and adjuvant CpG).

RNA involved in cell division, differentiation, growth, senescence, and apoptosis can also serve as an element for SNA.^[92,93,101,102]^ Multivalent RNA–AuNP conjugates passivated by PEG or modified with methyl groups are stable and functional.^[93]^

#### 3.3.2 | Modified nucleic acids

In addition to DNA or RNA, phosphoramidite chemistry enables various modifications to the nucleic acid chain on SNAs. Peptide nucleic acids (PNAs) and phosphorodiamidate morpholino oligonucleotides are entirely neutral nucleic acid analogs of DNA/RNA.^[103,104]^ They afford stronger hybridization, more excellent stability, and higher specificity in base pairing relative to negatively charged DNA.^[105,106]^ In 2006, the metallization of PNA with Pt nanoparticles by chemical binding, reduction, and deposition was reported for the first time.^[40]^ A recent PNA–miRNA–SNA sandwich detector showed a large number of adsorbed functional electroactive labels, minimizing background noise and achieving signal amplification with much higher sensitivity and specificity.^[107]^ Another form of modification is to increase the binding strength and stability of the conjugate by locked nucleic acids (LNAs), a class of nucleic acid analogs that contain a methylene bridge connecting the 2' oxygen and 4' carbon in the ribose moiety.^[108]^ By adding only four LNA bases to the particle sequence, the knockdown rate was increased by 66.6% in the target cells.^[8,109]^ Moreover, LNA–AuNP conjugates can increase their melting temperature by approximately 3°C, which allows for higher selectivity in detection schemes.^[110]^ Grafting active proteasomes onto the surface of SNAs also results in materials with relatively high stability.^[100]^

Although modifying SNAs by changing their surface nucleic acid aggregation is highly intriguing, many questions exist. Is there a theory that we can use to predict how SNAs, whether inorganic or organic core-based, transport in living organisms? How densely coated DNA/RNA strands differ from their linear counterparts remains unanswered. As shown by Mirkin et al., protein corona may play an essential role in regulating their biological interfacing. In addition, the metabolic fates of nucleic acids on the surface, in many cases, were found to be different from their core materials. Is it caused by the degradation of surface nucleotides or is there a nucleotide releasing mechanism? Answering these questions may help researchers control and program SNAs for biosensing, imaging, and disease theranostics.

## 4 | SNAs FOR DISEASE DIAGNOSIS AND TREATMENT

SNAs are highly attractive in the fast-growing field of nanomedicine, offering novel means to deliver nucleic acids. As our understanding of SNA deepens, the role of SNAs has shifted from simple nanosized vehicles for DNA/RNA delivery to nanosystems playing influential regulatory roles in cells and animals. After routine administration into mammals, SNAs can be recognized by Class A scavenger receptors expressed widely on the cell surface in circulation and endocytosed via a lipid-raft-dependent, caveolae-mediated pathway.^[21,111,112]^ Once inside the cell, the recognized SNAs bind with the corresponding target sequences with higher affinity and low toxicity, modifying gene expression directly. This may be harnessed for in vitro biosensing, disease imaging, gene therapy, drug delivery, and immunomodulatory therapy (Table 1).

### 4.1 | In vitro biosensing

**FIGURE 5:**
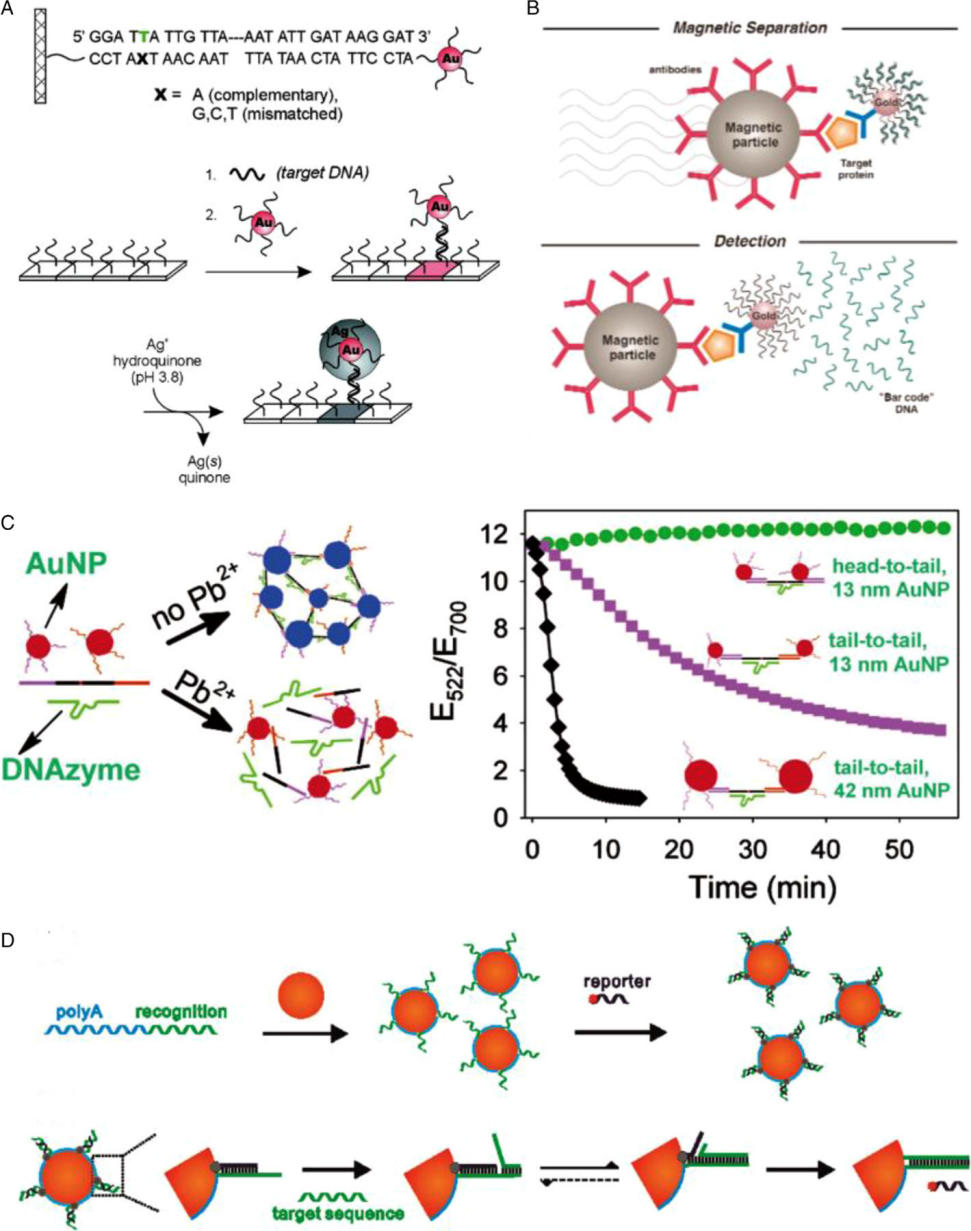
(A) The scanometric DNA array detection. Reproduced with permission: Copyright 2000, The American Association for the Advancement of Science.^[153]^ (B) An ultrasensitive method for detecting protein analytes relying on a sandwich of SNA, magnetic microparticle probes with antibodies, and the captured target. Reproduced with permission: Copyright 2003, The American Association for the Advancement of Science.^[172]^ (C) Improvement of a colorimetric lead sensor based on the assembly of gold nanoparticles by a Pb^2+^-dependent DNAzyme. Reproduced with permission: Copyright 2004, American Chemical Society.^[114]^ (D) PolyA-mediated SNA assembly strategy. Reproduced with permission: Copyright 2019, Elsevier^[157]^

The reversible melting behavior of SNA in a narrow temperature range can produce corresponding hybridization-dependent optical changes and may serve as a highly selective detection platform for in vitro molecular diagnosis.^[6]^ One of the most primitive applications of *in vitro* bioassays is colorimetric detection, where the target entity is captured to trigger reversible aggregation of the SNA probe, producing a visible red to purple color transition based on SPR effects.^[34,169,170]^ This process can be monitored by optical devices that distinguish components with mismatching nucleic acids on the SNA surface.^[120,121]^ Based on this, the DNA on the SNA surface is further replaced with DNase, which catalyzes specific hydrolytic cleavage in the presence of metal ions, therefore preventing aggregation from producing a red color, and the intensity of which reflects the metal ion levels in a concentration-dependent manner (Figure 5C).^[113–115]^ In principle, colorimetric detection systems designed with appropriately functionalized SNA conjugates can be used to detect any target that can affect the reversible melting behavior of SNAs. In addition, other systems rely on physiochemical changes of the inorganic SNA cores, such as magnetic resonance, raman spectrum, or fluorescent signal have been developed.^[20,122,155,171]^

Since 1998, colorimetric detection systems based on SNA conjugates have been used for quantitative measurement of nucleic acids,^[123,124,153,169]^ proteins,^[130,133,136,155,173]^, and metal ions (such as Pb^2+^,^[113–115]^ Hg^2+^,^[116,117]^ Cu^2+^,^[118]^, and Mg^2+^,^[119]^ Figure 5A). In 2000, a chip-based scanning assay similar to the conventional ELISA assay was developed.^[153]^ In 2003, SNA-related biological barcode analysis was invented to detect antigens and approved by the US FDA. The SNA with the “barcoded” DNA strand and the magnetic microparticle probe with antibodies simultaneously bind to the target detection antigen, and the barcoded DNA is shed from the probe, then the signal amplification is achieved via scanning the detection. By replicating barcode DNA via the polymerase chain reaction (PCR), the signal can be further amplified to six orders of magnitude (~3 pmol), more sensitive than conventional detection methods (Figure 5B).^[155,172]^ In 2009, the original team developed another microarray-based scanning immunoassay using antibody-modified SNA nanoparticle coupling technology. It was highly sensitive in detecting prostate-specific antigens and highly selective in detecting three protein cancer biomarkers at low picomolar concentrations in buffers and 10% serum.^[136]^ Later in 2010, a copper-catalyzed click chemistry method was used to link the acetylenic and azide groups at the ends of oligonucleotide chains on two SNAs for colorimetric detection of Cu^2+^.^[118]^ Subsequently, several studies explored the detection performance of DNA–AuNPs by optimizing the SNA structures and improving the SNA self-assembly. More sophisticated versions of SNA-coupling assays and detection methods have been created to achieve direct, rapid, sensitive, and specific detection, such as modulated toehold exchange SNAs,^[125]^ FNA-based SNAs,^[81,131]^ and SNAs with aligned carbon nanotube.^[126]^ New methods involved, including novel single nanoparticle, inductively coupled plasma mass spectrometry (SP-ICPMS) DNA assays,^[127]^ CRISPR-based diagnostics (CRISPR-Dx),^[128]^ and colorimetric protease assays with modular combinations of protease-responsive transcripts and SNAs.^[135]^

MicroRNAs (miRNAs) are considered as therapeutic targets and biomarkers for diseases like tumors and acute myocardial infarction (AMI). The use of SNAs to detect various disease-associated miRNAs has been extensively researched, and it has been challenging to directly and quantitatively analyze miRNAs in complex media, such as human serum.^[174]^ By using polyA-mediated SNAs, a detectable fluorescent signal is generated by regulating the length of polyA on bound SNAs, which displace the reporter upon binding to the target sequence.^[84]^ The multicolor detection of three different miRNAs associated with pancreatic cancer was achieved, making the detection method more flexible and controllable (Figure 5D).^[157]^ A later study invented erythrocyte membrane-biology interface SNAs for miRNA detection, which enables customized signal amplification and exhibits interference-resistant performance for single-step quantification of miRNAs in complex media.^[158]^ Within the LSNA, hydrophilic QDs, hydrophobic QDs, and AuNPs are able to mix in a site-selective manner, constituting a liposomal “core-resonance energy transfer” system surrounded by an SNA shell (Figure 3C), for colorimetric amplified detection of miRNAs through an auxiliary biochemical reaction.^[49]^ Li et al. established an electrochemiluminescence sensing platform for circulating miRNAs using AuNPs@G-quadruplex SNA enzyme (SNAzyme) as a nanocatalyst, with a portable smartphone as a detector to visualize AMI-associated miRNAs in actual patient serum for the first time.^[160–162]^ Wei et al., developed an enzyme-free SPR imaging based on a catalytic hairpin device and SNA to enable differentiation of single-base differences and members of homologous miRNA families.^[159]^

**FIGURE 6:**
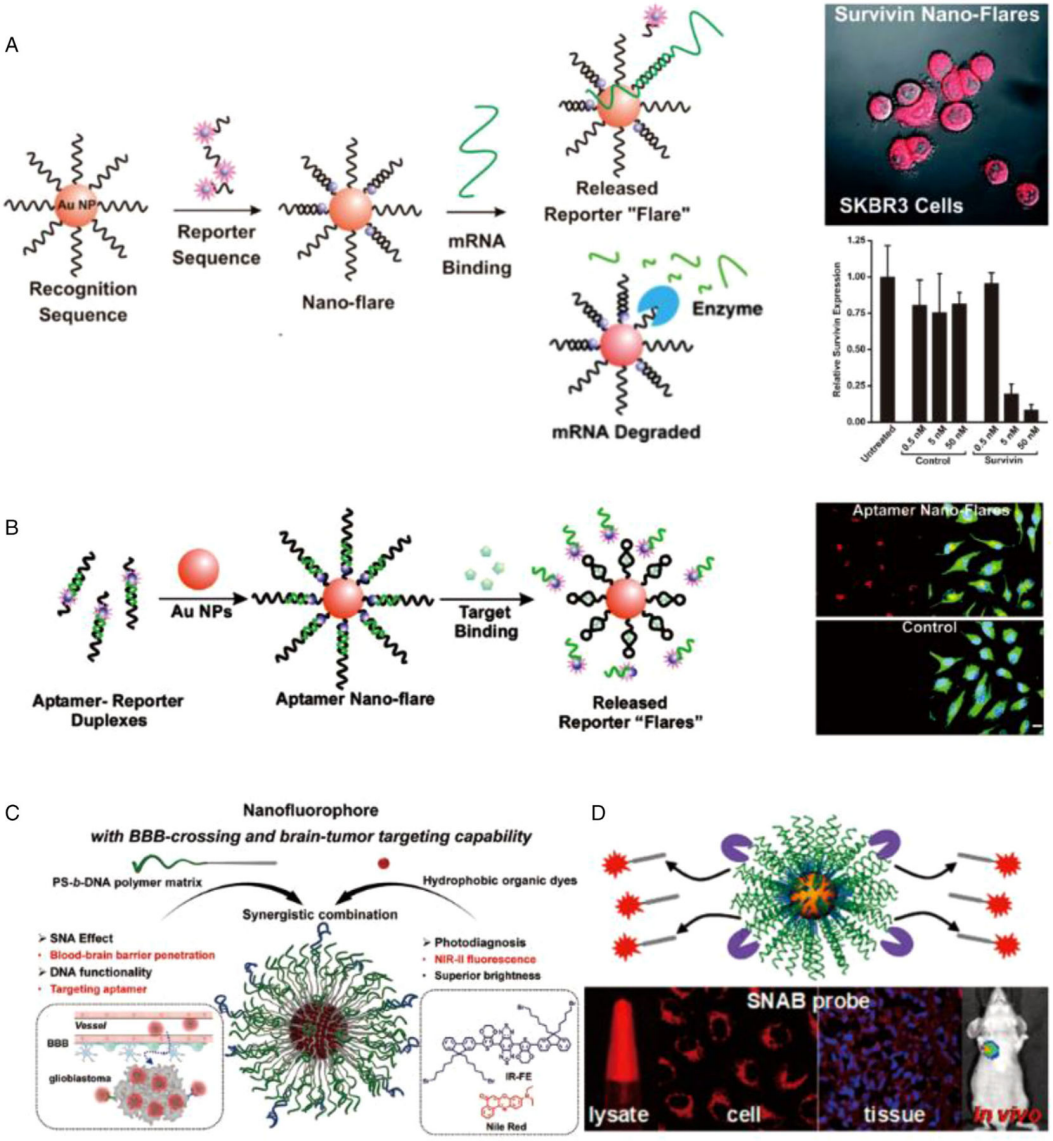
(A) “Nanoflares” for detecting mRNA in living cells. On this basis, an SNA was developed that can simultaneously detect and regulate mRNA. Reproduced with permission: Copyright 2007, American Chemical Society.^[15]^ Copyright 2009, American Chemical Society.^[138]^ (B) An aptamer nanoflare can directly quantify an intracellular analyte in a living cell. Reproduced with permission: Copyright 2009, American Chemical Society.^[165]^ (C) An amphiphilic DNA block copolymer PS-b-DNA was synthesized and used as a polymer matrix to fabricate a NIR-II-emitting nanofluorophore, breaking through the limit of the blood–brain barrier for brain-tumor imaging. Reproduced with permission: Copyright 2020, Wiley.^[175]^ (D) Combining SNAs with finely designed molecular beacons (SNA beacons, dubbed SNAB technology) enables the detection of tumor cells on multiple platforms. Reproduced with permission: Copyright 2018, American Chemical Society^[132]^

In addition to detecting miRNAs, SNAs have been applied in single nucleotide polymorphism identification, such as detecting HIV-1 viral nucleic acid and coronavirus SARSCoV-2.^[129,154]^ The rapid and straightforward SNA biosensor is comparable to real-time PCR methods in terms of sensitivity and is vital for controlling disease pandemics. SNA biosensors have been reported to successfully assess telomerase activity for the early diagnosis of malignancies in detecting proteases (Figure 6D).^[130–132]^ In addition, the colorimetric protease assay mentioned above can sensitively assess biomarker proteins such as matrix metalloproteinase-2 (MMP-2), thrombin, and hepatitis C virus NS3/4A in biological and clinical samples.[135] This colorimetric method can be made into test paper and detected by portable phones, which has a vast and promising prospect.

### 4.2 | Intracellular assessment

In addition to in vitro metal ion and biomarker detection, SNA readily and autonomously enters cells, resists degradation by intracellular endonucleases, and binds target sequences with high specificity targeting, allowing for efficient intracellular diagnostic and imaging of small molecules.^[94]^ In contrast to essential tools often applied to quantify the activity of living systems such as in situ staining, molecular beacons, and fluorescence resonance energy transfer, SNA does not require transfection agents and is intracellularly stable with low background signal. “Nanoflare” (NF) is one of the most successful commercial applications of SNA technology, which uses a fluorescent dye-labeled “reporter” sequence to bind to SNA (five times faster than single-stranded SNA-gold nanoparticle conjugates), showing low fluorescence due to quenching of the gold core.^[15]^ Once the SNA binds to the target mRNA, the reporter nucleotide is displaced to show high fluorescence.^[137]^ In confocal microscopy and flow cytometry of living cells, NFs can theoretically quantify any mRNA sequence (Figure 6A and B).^[15,138,143,144,165]^ As such, multiplexed NFs obtained by hybridization of reporter sequences with different fluorophore labels with SNA are able to detect two different mRNA target sequences in living cells simultaneously.^[139]^ In addition, NFs can also be used for intracellular evaluation by synthesizing nuclear magnetic resonance (NMR) probes via replacing the fluorophore with NMR-active fluorine-19 (19F)-modified 5-fluorouracil nucleobase sequences.^[145]^ By transferring fluorophore-bound reporters to transcripts to identify target RNA transcripts in a sequence-specific manner, a novel sticky-flare can track RNA transport throughout the cell by fluorescence microscopy.^[140]^ The creation of aptamer NFs marks the first time SNA can detect the arrival of material elements other than DNA and RNA, including ions, small molecules, and proteins (Figure 6D).^[132,164,166,176]^ For example, ATP-targeted aptamer nanoflares (ATP-ANFs) are initially quenched, where the ATP aptamer changes its conformation in the presence of ATP after binding, exposing the signal sequence and leading to a significant increase in fluorescence.^[165]^ In 2018, NF technology was used to measure connective tissue growth factor (CTGF) as a visual indicator of hypertrophic scars and keloids. Topical application of NFs allows visual and spectral quantification of abnormal scar cells in the skin (such as mouse/rabbit ears and living human skin models).^[177]^ It provides an effective tool for noninvasive and accurate clinical diagnosis of scar types and severity and clinical treatment decisions. In 2020, organic SNAs designed using polystyrene-*b*-polyethylene glycol (PS-*b*-PEG) can be delivered across the blood–brain barrier, enabling noninvasive imaging of glioblastoma (GBM) (Figure 6C).^[175]^ In recent years, many NF-based SNA probes have been developed for intracellular evaluation, such as SNA and graphene oxide composite probes (AuNP/GO probes) to identify and monitor cytoplasmic targets miRNA (pre-miRNAs) and mature miRNAs in live cells in situ.^[141]^ The “sandwich” electrochemical exosome miRNA sensor (SEEmiR) detects miRNAs in breast cancer patients.^[107]^ DNA/RNA duplex crown nanoprobe assays for intracellular RNase H activity.^[146]^ Nanoamplicon comparator probe has the ability to monitor miRNA expression levels in different cell lines under external stimuli.^[142]^

However, several limitations of the NF technology remain: NFs are susceptible to nuclease degradation, reporter strand dehybridization, and interference from biothiol groups that cleave the gold-thiol linkage and produce false-positive signals.^[178,64]^ In addition, the binding kinetics to the target on the SNA may be affected by the displacement of NFs.^[179]^ Also, NFs can only be designed for targets with known nucleic acid recognition sequences, limiting their broader application.^[180]^

Several techniques have also been investigated to improve the limitations of NF technology.^[87,181]^ Relying on pretargeting based on nucleic acid hybridization, positron emission tomography (PET) probes for sensitive and specific imaging of tumor tissue were synthesized by injecting mouse breast cancer models with SNAs.^[182]^ In 2020, Pro-SNAs were also used for live cell analysis, and their modular structure makes it easy to change the protein core and programmable nucleic acid shell. Pro-SNAs with glucose oxidase as the functional protein core overcome the limitations associated with traditional fluorophore/quencher-based gold NFs and have been shown to detect relative changes in pH and glucose concentration in living cells.^[64]^ SNA surface probes can be designed to provide unlimited intracellular molecular targets and many possible labeling and readout strategies that can be applied to a variety of imaging techniques, including fluorescence phenomena, computed tomography, MRI, and PET.

### 4.3 | Gene regulation

SNAs can be used as intracellular gene regulators to suppress transcription and translation of overexpressed genes, controllably enabling gene knockdown while controlling the expression of intracellularly relevant proteins.^[94,183,184]^ In this process, SNAs deliver short interfering RNA (siRNA) or antisense oligonucleotides into the cell via endocytosis, where it binds to the RNA-induced silencing complex (RISC).^[96,185,186]^ Upon double-stranded uncoupling, the strand bound to RISC directs its complementary adhesion to the target mRNA strand, initiating its degradation and translational repression for gene regulation.^[138]^ The knockdown effect of SNAs is more durable at the mRNA and protein levels than cation-delivered nucleic acids.^[96]^ In 2006, antisense SNAs were first employed as viable intracellular regulators with the successful downregulation of green fluorescent protein.^[94]^ Since then, research applications using SNAs to transport genes and control the expression of target proteins have mushroomed. SNAs have been used to target a variety of genes including HER2,^[167]^ Bcl2,^[10,19,148,187]^ ganglioside GM3 synthase,^[98]^ epidermal growth factor receptor (EGFR),^[13]^ Malat1,^[28]^ O6-methylguanineDNA methyltransferase (MGMT),^[12,168]^ and TNF-*α* and IL17RA.^[11]^

**FIGURE 7:**
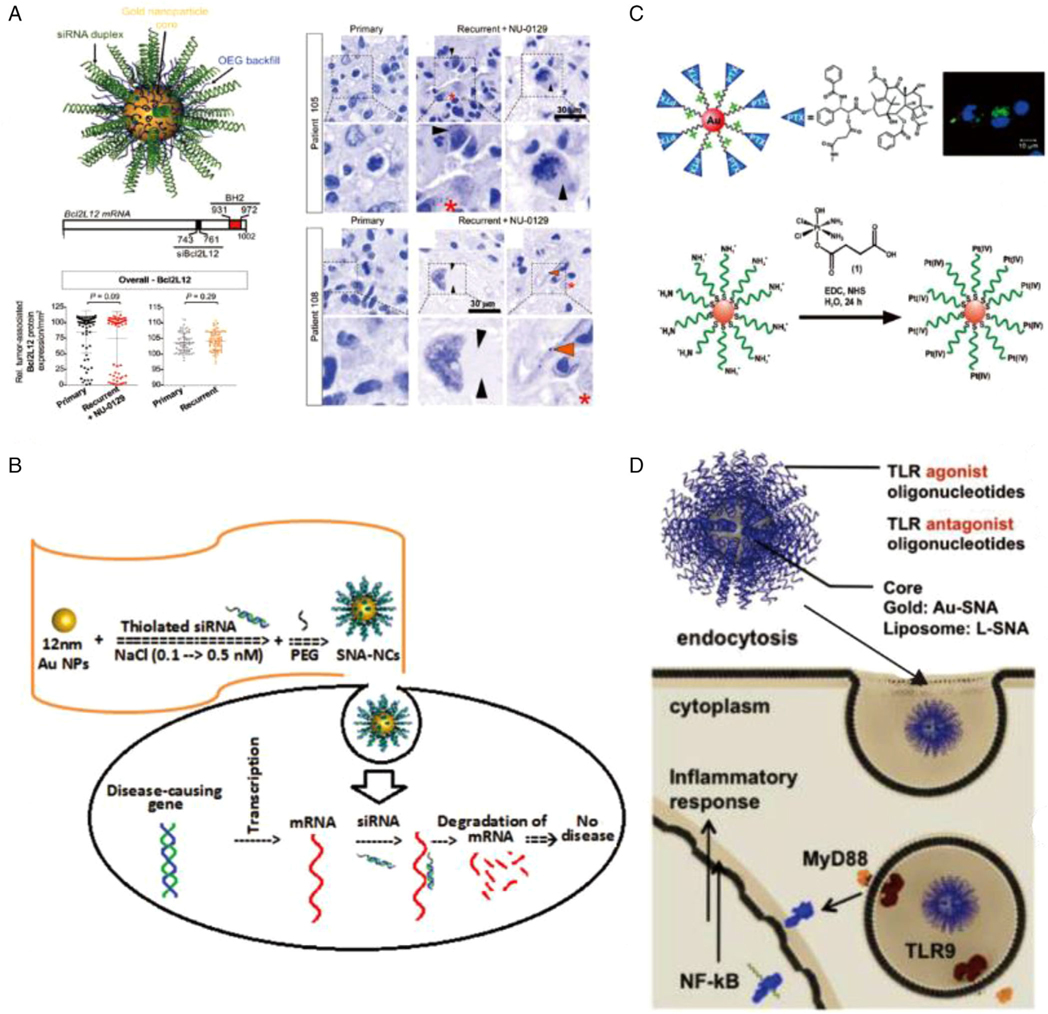
(A) The first-in-human phase 0 clinical study of RNA interference-based SNAs in patients with recurrent GBM. Reproduced with permission: Copyright 2021, The American Association for the Advancement of Science.^[19]^ (B) SNA nanoparticle conjugates for gene regulation in psoriasis. Reproduced with permission: Copyright 2017, Elsevier.^[188]^ (C) Multimodal drug delivery systems are siRNA-based that covalently link paclitaxel molecules or cisplatin prodrugs to gold nanoparticles via fluorescent/amine-functionalized oligonucleotide linkers with imaging and enhanced therapeutic capabilities. Reproduced with permission: Copyright 2011, American Chemical Society.^[21]^ Copyright 2009, American Chemical Society.^[151]^ (D) Immunomodulatory SNAs for stimulating (immunostimulatory, IS-SNAs) or regulating (immunoregulatory, IR-SNAs) immune responses have been internalized by the immune. Cells. Reproduced with permission: Copyright 2015, National Academy of Sciences^[51]^

Dermatological diseases and neuroblastoma are the two main areas where local delivery of SNA for gene therapy is currently available, and the relevant content has entered different clinical trials (Figure 7A).^[19]^ In dermatology, SNA has excellent gene therapy effects for skin inflammation, skin tumors, dominant-negative hereditary skin diseases, and other related disorders.^[177]^ Among them, the most promising clinical translational application is in psoriasis (Figure 7B).^[188]^ SNAs have been shown to be efficacious in penetrating uniformly thickened skin, knocking down gene targets, and reversing skin disease in mouse and human three-dimensional culture models of psoriasis.^[13,95]^ Phase I clinical trials have shown the safety and promise of SNAs as topical agents against TNF-*α* and interleukin 17 receptor A (IL17RA), the inhibitory targets against moderate to severe psoriasis.^[11]^ The two drugs involved are named AST-005 and XCUR17, respectively. In patients with mild to moderate psoriasis, both new drugs were well-tolerated with no adverse effects. AST-005 resulted in a significant reduction in TNF-*α*, while XCUR17 had no IL17RA knockdown but reduced gene expression of K16 and inflammation.^[95]^ This suggests a very promising clinical pathway for SNA-based local treatments.

In the treatment of glioblastoma (GBM), an aggressive form of brain cancer, SNAs have been shown to penetrate the blood–brain barrier and improve survival in mice.^[10,189]^ Studies have demonstrated that SNAs can directly cleave MGMT mRNA associated with chemoresistance in GBM and increase the susceptibility of GBM cells to treatment-mediated apoptosis.^[168]^ Mirkin and colleagues developed the first drug to use SNAs for systemic therapy in humans, approved as an investigational new drug named NU-0129 through the US FDA. NU-0129 delivers siRNA targeting Bcl2L12 with preclinical toxicology study in nonhuman primates and the first human phase 0 clinical trials in eight GBM patients (NCT03020017) completed.^[19]^ All animals survived the scheduled necropsy with no observed adverse effect level but varying degrees of NU-0129–related clinical observations of purple or blue discoloration of va rious body surfaces. The clinical trial declared that systemic administration of NU-0129 was safe, resulted in uptake of nanoconjugates by endothelial cells, immune cells, and tumor cells, and was associated with reduced expression of target proteins in patients’ GBM tumors. Furthermore, SNAs have a role in the therapy of lung cancer by regulating lncRNAs in cells to target nuclear retention of knockdown of metastasis-associated lung adenocarcinoma transcript 1 (Malat1), as well as upregulate tumor suppressor messenger RNAs associated with Malat1 knockdown.^[28]^

### 4.4 | Drug delivery

The stability of the nucleic acid shell allows for the attachment and delivery of other chemical agents. Therefore, SNA can be applied as a versatile platform for chemotherapeutic drug delivery.^[27]^ The oncology chemotherapeutic agent platinum(IV) prodrug has been covalently linked to AuNP-based SNA, creating an efficient delivery vehicle for cisplatin (Figure 7C).^[21,171]^ Once internalized by the cell, the platinum(IV) complex is reduced to the cytotoxic Pt(II) substance and released into the cytoplasm. Insoluble and difficult-to-deliver drugs like paclitaxel achieve superior therapeutic efficacy by binding to the SNA shell with high stability and cellular uptake.^[75]^ SNA promotes apoptosis of primary patient chronic lymphocytic leukemia (CLL) lymphocytes by encapsulating BKM120, an anticancer drug for CLL, and acts as a sensitizer for other antitumor agents.^[152]^ Dox-PSNA loaded with adriamycin, which targets cervical cancer cells SOV3, exhibits comparable cytotoxicity to the free drug.^[22]^

In addition to the above drugs, several studies have successfully demonstrated the effective delivery of diagnostic and therapeutic nucleotides and model drugs.^[74,112,190,191]^ Gene targets and drug targets can be obtained through SNAs without the need for an adjuvant vector system. A recent study reported that an SNA structure containing an antisense oligonucleotide and a tyrosinase inhibitor prodrug both reduced melanin content in B16F10 melanoma cells and exhibited potent antimelanogenic effects in UVB-induced pigmentation.^[150]^

Extensive exploration of the mechanism of SNA distribution in the organism and methodological improvement studies hold promise for further improvement of SNA delivery efficiency and efficacy (Figure 7C).^[21,46,57,61]^ One study greatly simplified the SNA drug delivery system by assembling only four strands while minimizing the SNAs to load and release polymer couples of nucleic acids. Two “pillar” amphiphilic chains were hybridized with “bridge” chains, and oligonucleotide therapeutics were assembled with horizontal portions of the bridge chains.^[192]^ PLGA-SNAs are used to encapsulate hydrophobic model drugs and provide control over the release kinetics of the encapsulated cargo in the context of the SNA platform.^[72]^

### 4.5 | Immune-modulation

The use of SNAs for immune-modulation has been increasingly studied in recent years. SNAs can be used as sequence-specific, potent, and therapeutically meaningful constructs and are superior to linear nonlipid phosphodiester DNAs.^[48,51]^ The high biocompatibility and modular design allow SNAs to bind to other functional components (e.g., anti-PD-1 antibodies, RNA, TLR ligands) to deliver multiple components to a single cell, and this flexibility is of great value for improving immunotherapy.^[56]^ The lipid tail of the DNA amphiphiles (CpG for TLR-9 stimulation) was inserted into the hydrophobic region of Pluronic F127 micelles to obtain TLR-9 immunomodulators efficiently.^[73]^ The most representative new drug is AST-008, an LSNA TLR-9 agonist, which is used to stimulate or modulate macrophages and antigen-presenting cells by carrying immunomodulatory oligonucleotides or antigens recognized by TLRs to induce immune responses in Merkel cells and cutaneous squamous cell carcinomas for the treatment of various skin cancers (Figure 7D).^[51]^ Relevant clinical applications have been conducted in phase I/Ib human trials (NCT03086278), and a phase II trial is ongoing. Completed trials have shown no serious adverse events, mild injection site reactions and flulike symptoms, dose-related systemic immune activation, and more diverse tumor cell infiltration with AST-008 injection compared to noninjected tumors.^[95]^ As such, additional immunostimulatory SNAs consisting of RNAs selective for TLR-7/8 were synthesized and characterized.^[50]^

SNAs can be designed to deliver multiple antigenic proteins in combination with immunostimulatory CpG oligodeoxynucleotides to antigen-presenting cells to induce robust immune responses to diseases such as influenza and dengue fever.^[55]^ In addition, this can be enhanced by designing SNAs to co-deliver various adjuvants, small molecule immune potentiators, or antigenic proteins. Immunostimulatory nucleic acids or stimulators of interferon genes (STING) agonists can also be combined with SNAs with current immunotherapies to modulate the tumor microenvironment and identify novel and effective methods of therapeutic combinations. The latest hot spot focuses on the development of SNA vaccines, where high-throughput synthesis and analysis have identified a structure with superior performance as a therapeutic vaccine in several animal models.^[23,53]^ A therapeutic vaccination strategy against prostate cancer has been developed and is available for clinical use (Sipuleucel-T).^[193]^ On this basis, immunostimulatory SNA (IS-SNA) nanostructures consisting of CpG oligonucleotides as adjuvants and prostate cancer peptide antigens were developed to induce superstrong cytotoxic T lymphocyte-mediated target cell antigen-specific killing by increasing codelivery to DCs.^[23,194]^

## 5 | CHALLENGES OF SNAs FOR IN VIVO APPLICATIONS

Despite the proven potential of SNAs for biomedical applications in biosensing, bioimaging, and biomedicine, significant issues remain unanswered and warrant further investigation. First and foremost is the examination of SNA stability at all levels such as intracellular, intratissue, as well as across organs. Studies have confirmed increased DNA stability against DNases using the most classic AuNP-based SNAs. However, a more in-depth evaluation of SNA stability, both the core material and surface oligonucleotides, is highly desired.

Second, the detailed biological profiles of SNAs in living organisms are not yet fully understood. Animal studies showed tumor uptake of AuNP-based SNAs with a strong background from the liver and spleen. Factors affecting SNA’s biodistribution are far from clear. Current studies primarily focus on their shape and size. Other possible parameters worth investigating include (1) SNA density as determined by the core materials, since inorganic core-based SNAs generally have a higher density than their inorganic counterparts; (2) surface protein corona as determined by nucleic acid sequence and their interaction with blood serum; and (3) surface nucleotide regularity as denoted by the length of DNA/RNA strands and their distribution density on the SNA surface.

Moreover, the biological interaction of SNA with the natural immune system is yet to be illustrated. Little to almost none is understood on how these “furry” nucleic acid aggregates may be processed and metabolized in vivo. As for their close relative, FNAs were found to have a quick blood elimination and improved kidney excretion/accumulation after intravenous injection.^[195,196]^ The answer to what might cause their differences in nano-bio interfacing may help researchers design and program SNA with better biological profiles in vivo.

## 6 | CONCLUSION AND FUTURE PERSPECTIVES

In summary, SNAs serve as a library of different core materials and programmable surface DNA/RNAs for biosensing, bioimaging, and biomedical applications. The core materials not only serve as the scaffold for surface nucleic acids coating but also bring intrinsic optical, physical, and chemical properties to the final SNA product. On the other hand, surface nucleic acids are modified and designed following the application and play pivotal roles in determining the biological functions of the formed SNAs.

When used for in vitro biosensing, SNA has evolved from a colorimetric examination of metal ions to the detection of nucleotides, nucleases, proteases, and specific proteins associated with tumors or diseases (such as AMI).^[160–162]^ For in vivo bioimaging, the classic NF has been expanded to the development of sticky flares, two-photon nanoprobes, and the implementation of radionuclide ^19^F-labeled SNA imaging. The detection range has also been extended from nucleic acids to important biomarkers such as CTGF, ATPases, and glucose. Gene regulation is another focus of SNA. Advancing toward clinical translation, AST-005, AST-008, XCUR-17, and NU-0129 have been reported to cure skin diseases such as pruritus, psoriasis, and GBM tumors, whereas increasing studies on breast, lung, head, and neck cancers are underway. Moreover, as a superior drug delivery vehicle, SNAs can carry therapeutic nucleotides, chemotherapeutic agents, and immunosuppressants.

To date, research on SNAs ranges from in vitro biosensing to in vivo biomedicine, and the AuNP-based SNA has found its way into a phase 0/1 clinical trial, marking a substantial success for SNA’s further translation. Given the almost infinite combination of nanomaterials and nucleic acids that have been and can be used for SNA preparation, the research field of SNA will continue to thrive, promising many unforeseen applications to be discovered. The underlying possibilities of designing, predicting, and programming SNA’s biological behaviors after we reveal their biointerfacing in vivo will provide a ready route to an almost infinite variety for disease theranostics.

## Figures and Tables

**TABLE 1:** SNAs for disease diagnosis and treatment

**Types of core**		**Advantage(s)**	**Liimtation(s)**	**In vivo/in vitro**	**Application**	**Features**
Inorganic cores	AuNP	• Ease of synthesis • Wide range of particle diameters • Facile surface modification • Unique optical properties	• Long-term cytotoxicity • Immune response • Unclear pathway of cellular uptake	In vitro	Biosensing	• Metal ions^[113–119]^ • Complementary DNA^[34,81,120–128]^ • HIV RNA^[129]^ • SARS-cov-2 RNA^[154]^ • Enzyme: telomerase^[130,131,132]^, DNase I^[133,134]^, MMP-2^[135]^, • Prostate cancer: PSA^[136]^ • mRNA of skeletal stem cells^[137]^
					Intracellular assessment	• mRNA^[138–140,15]^ • miRNA^[141,142]^ • Complementary DNA^[143–145]^ • RNase H^[146]^
					Gene regulation	• Breast cancer: Survivin^[147]^ • Glioblastoma: Bcl2L12^[10,148]^ • Prostate cancer: miR-21, PTEN^[149]^ • Skin disease: EGER, ERK^[13]^ • Melanoma: melanocortin 1 receptor^[150]^
					Drug delivery	• Melanoma: tyrosinase inhibitor prodrugs^[150]^ • Ovarian cancer: paclitaxel^[21,75]^ • Cervical cancer/lung cancer: platinum (Pt)^[151]^ • Camptothecin^[112,74]^
					Immune-modulation	• Breast cancer: CpG-1826^[52]^ • Nonalcoholic steatohepatitis TLR-3/7/8/9^[51]^
				In vivo	Gene regulation	• Glioblastoma: MGMT,^[12]^ Bcl2L12^[19]^ • Diabetes: GM3 synthase^[98]^
					Drug delivery	• chronic lymphocytic leukemia/cervical cancer: BKM120^[152]^
	AgNP	• Antimicrobial properties • High electrical conductivity • Optical properties		In vitro	Biosensing	• Complementary DNA^[20,153]^
	Magnetic microparticles-NP	• Magnetic-related applications • Orientation by controlling the external magnetic field		In vitro	Intracellular assessment	• Prostate cancer: PSA^[155]^
	QDs	• Exclusive quantum confinement effect	• Potential toxicity	In vitro	Intracellular assessment	• C166 cells^[46]^
Organic cores	LSNA	• Ease of self-assembly • Biocompatibility • Chemically adjustable structure • Carrying large drug payloads	• Low solubility • Short half-life • Instability	In vivo	Gene regulation	• Lung adenocarcinoma: Malat1 (lncRNA)^[28]^ • Psoriasis: TNF-*α* and IL17RA (siRNA)^[11]^
					Immune-modulation	• TLR-7/8^[50]^
	Pro-SNA	• Biodegradable, biocompatible • Efficient intracellular delivery and transfection • Multiple synthesis methods	• Difficulty of scale-up • Thermodynamic instability • Protein strain potential exists^[156]^	In vitro	Intracellular assessment	• Glucose^[64]^
	DBBC-based micelle	• Higher nucleic acid surface density, more cooperative thermal denaturation properties, and more efficient transfection-free cellular uptake compared to AuNP-based • Capability of assembling into spherical micelles	• Lack of manufacturing paradigm • Immune response	In vitro	Gene regulation	• EGFP^[67]^
	PLGA	• FDA-approved polymer • Biodegradable • Biocompatible • Easy formulation of drug-carrying devices	• Prevention of protein deactivation • Aggregation during encapsulation	In vitro	Drug delivery	• Coumarin 6^[72]^
	Pluronic F127-based micellar	• Easily assembled and stable • A wide range of hydrophobic cargos	• Fast degradation	In vitro	Immune-modulation	• TLR-9^[73]^
	Dox-containing PNPs	• Ease of synthesis • High stability • Sustained and extended drug release profile	• Toxic degradation • Toxic monomers aggregation • Inadequate degradation	In vitro	Drug delivery	• Cervical cancer: DOX^[22]^
**Types of surface**		**Advantage(s)**	**Limitation(s)**	**In vivo/in vitro**	**Application**	**Aspect(s)**
PolyA-mediated		• High cell entry efficiency • Insensitive to the configuration of the anti-miRNA sequences • Programmatically adjust the valence of SNA	• Accurate control of poly(A) lengths is challenging	In vitro	Biosensing	• miRNA^[157]^
Aptamer (G-quadruplex)		• Nonimmunogenic • Thermodynamically and chemically stable • Nuclease stability • Superior affinity and specificity to the target • Better stability, and higher reproducibility	• Nonspecific interactions • Limited bioavailability • Quick degradation	In vitro	Biosensing	• miRNA^[160,162]^
					Drug delivery	• CLL/Cervical cancer: TMPyP4^[163]^
				In vitro	Intracellular assessment	• K^+[164]^ • ATP^[165]^ • Cytc^[166]^
PNA		• Stronger hybridization, greater stability, and higher specificity in base pairing compared to negatively charged DNA • Extended lifetime both in vivo and in vitro	• Poor water solubility • Poor penetration through the cell membrane	In vitro	Biosensing	• Exosomal miRNAs^[107]^
Antibody		• Increased sensitivity and selectivity	• High cost	In vitro	Gene regulation	• Breast cancer: HER2^[167]^
Ribozymes		• Specifical binding and clavation of an mRNA substrate	• Complex synthesis • Low efficacy	Invitto	Gene regulation	• Glioblastoma: MGMT^[168]^
